# Omega-3 fatty acids coordinate glucose and lipid metabolism in diabetic patients

**DOI:** 10.1186/s12944-022-01642-w

**Published:** 2022-03-25

**Authors:** Pasquale Mone, Fahimeh Varzideh, Urna Kansakar, Carmine Infante, Angela Lombardi, Antonio de Donato, Salvatore Frullone, Gaetano Santulli

**Affiliations:** 1 Department of Medicine - Einstein-Sinai Diabetes Research Center, Albert Einstein College of Medicine, New York, USA; 2 ASL Avellino, Avellino, Italy; 3 University of Campania “Luigi Vanvitelli”, Naples, Italy; 4 University of Naples “Federico II”, Naples, Italy

**Keywords:** Fish oil, Omega-3 fatty acids, PUFA, T2DM, Vegetable oils

## Abstract

Omega 3 polyunsaturated fatty acids (n-3 PUFA) are known to have beneficial effects on cardiovascular and metabolic health. However, whether different sources of n-3 PUFA, for instance fatty fish vs vegetable oils, could elicit different effects on glucose and lipid metabolism, remains to be determined. Herein we examine recent findings showing that while a plant-based n-3 PUFA supplementation for six months can reduce fasting blood glucose, marine-based n-3 PUFA can instead reduce serum levels of triglycerides. We also discuss the potential molecular mechanisms that could underlie these different effects on the regulation of glycolipid metabolism.

## Introduction

Type 2 Diabetes Mellitus (T2DM) is commonly associated with dyslipidemia, leading to a higher risk of atherosclerosis and cardiovascular diseases [1, 2]. Hypertriglyceridemia represents an important risk factor for atherosclerosis, especially in diabetic patients [3]. Henceforth, nutraceutical supplementations might help reduce the risk of adverse events and/or improving the quality of life of these subjects [4], in combination with improved lifestyle habits and pharmacological intervention, to prevent/delay the onset of cardiovascular complications.

Hyperglycemia and hyperlipidemia are known to strongly impact the pathophysiology of coronary artery disease, also by driving endothelial dysfunction [3, 5–7]. Moreover, endothelial dysfunction remains among the main mechanisms underlying the onset of cardiovascular adverse events and outcomes in people with Type 1 Diabetes Mellitus (T1DM) or T2DM [7–12]. A dietary intervention with 500 g/week of fatty fish, equivalent to ~ 1 g/day of omega 3 polyunsaturated fatty acids (n-3 PUFA), like eicosapentaenoic acid (EPA; 20:5n-3) and docosahexaenoic acid (DHA; 22:6n-3), have been shown to have a cardioprotective effect by inhibiting platelet-monocyte aggregation, and a higher dietary intake can also improve endothelial function [13].

## Discussion

**Fig. 1:**
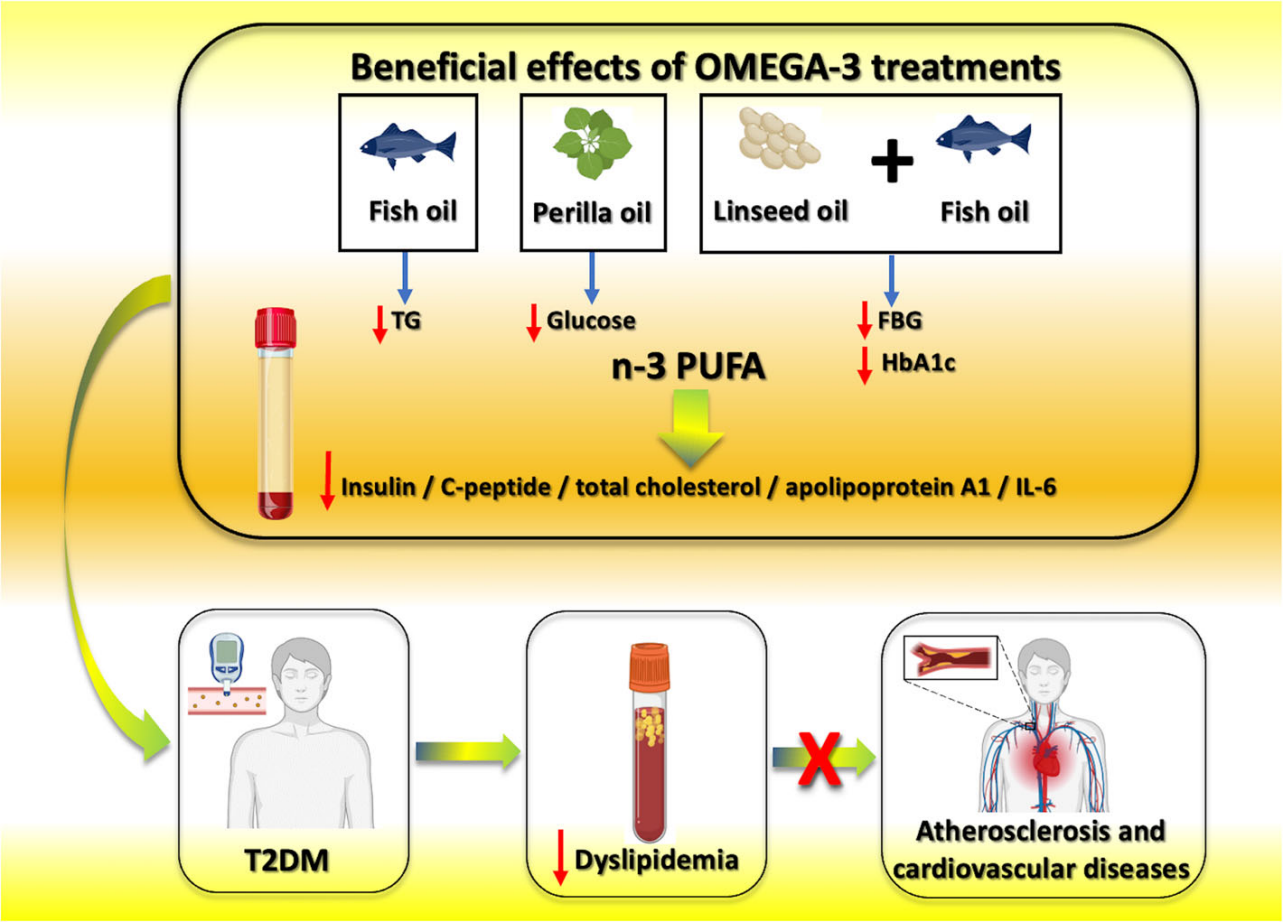
Different effects of marine-derived and plant-derived n-3 PUFA on lipid and glucose metabolism in people with T2DM. FBG: fasting blood glucose; Hb1Ac: glycated hemoglobin; IL-6: interleukin-6; n-3 PUFA: long chain polyunsaturated fatty acids; T2DM: type 2 diabetes mellitus; TG: triglycerides. Some images have been created with biorender.com

The potential different effects of diverse sources of n-3 PUFA (e.g. fish vs vegetable [14, 15]) on glycolipid metabolism have not been fully investigated. A known difference between vegetable and marine n-3 PUFA is the cholesterol lowering effect vs triglyceride lowering effect, respectively; nevertheless, whether vegetable n-3 PUFA may have an effect on blood glucose has not been established [15–18]. In an elegant double-blind clinical trial, Liu and colleagues evaluated the different effects of marine-derived and plant-derived omega-3 PUFA on the fatty acids of erythrocytes and glycolipid metabolism in patients with diabetes [19]. The study was conducted on 150 patients with a diagnosis of T2DM, of which 52 were randomly assigned to the fish oil group, 50 to the perilla oil group, and 48 to the linseed and fish oil group. All patients were followed up for six months. Intriguingly, while the supplementation with perilla oil (a vegetable oil rich in alpha-linolenic acid [20]) significantly decreased fasting blood glucose compared to baseline, fish oil supplementation prompted a marked reduction of serum triglycerides (TG) levels. Therefore, marine-based and plant-based n-3 PUFAs exhibited different effects on the regulation of glycolipid metabolism (Fig. 1). Intriguingly, the administration of all types of n-3 PUFA significantly reduced insulin and C-peptide concentrations compared to baseline. Similarly, serum total cholesterol, apolipoprotein A1, and IL-6 levels significantly decreased in all the treatment groups compared to baseline values (Fig. 1).

These findings are noteworthy inasmuch as the association of diabetes mellitus and dyslipidemia is known to significantly increase the risk of cardiovascular complications [1, 21, 22], particularly coronary artery disease [23, 24]. Furthermore, the diverse impact on glucose and lipid homeostasis shown by the different sources of n-3 PUFA might help explain numerous controversial results in studies examining the effects on n-3 PUFA consumption in people with T2DM [14, 15, 25–29].

The molecular mechanisms underlying the different effects of plant-based vs marine-based n-3 PUFAs are not explored by the Authors and deserve further dedicated investigation. Potential mechanisms include the existence of different receptors for n-3 PUFA, which could trigger different glucometabolic responses. For instance, G-protein coupled receptor 120 (GPR120) is a functional receptor for alpha-linolenic acid [30] expressed on endocrine L-cells lining the gut which has been shown to directly mediate PUFA-induced increases in glucagon like peptide-1 (GLP-1) [31]. Other receptors activated by free fatty acids include GPR40, mostly engaged by long chain fatty acids, GPR84 engaged by medium chain fatty acids, and GPR41 and GPR43, engaged by short chain fatty acids [32–34]. An action on pancreatic islets (direct or mediated by GLP-1 [35–37]), or on hepatic and adipose tissue, represent other, not mutually exclusive possibilities. Supporting the latter hypothesis, marine n-3 PUFA have been shown to lower plasma levels of proprotein convertase subtilisin kexin type 9 (PCSK9) [38]; since PCSK9 inhibitors are used as a medication to reduce hypercholesterolemia, this finding could have major implications for CVD treatment [7, 39, 40].

The study by Liu and collaborators is not exempt from limitations. For instance, the sample size was relatively small. Additionally, many patients had a high body mass index and the mean systolic blood pressure at baseline was above 140 mmHg in all three groups, suggesting that most of the patients in the study were hypertensive. These aspects imply that the findings should not be extended to normotensive and non-overweight patients. Some concerns on the blinding process are mentioned by the Authors (“the assessors who gathered the information and analysts were not fully blinded”) but not better addressed. Therefore, further studies in larger populations, ideally not limited to T2DM patients, and with a longer follow-up are warranted. Nevertheless, the result of this clinical trial shed light on the importance of the source of n-3 PUFA in the evaluation of glucose and lipid metabolism.

## Data Availability

Not applicable.

## Abbreviations

n-3 PUFA: Omega-3 polyunsaturated fatty acid
EPA: Eicosapentaenoic acid, 20:5n-3
DHA: Docosahexaenoic acid, 22:6n-3
T1DM: Type 1 Diabetes Mellitus
T2DM: Type 2 Diabetes Mellitus
TG: Triglycerides
